# Intergenerational Transmission of Effects of Women's Stressors During Pregnancy: Child Psychopathology and the Protective Role of Parenting

**DOI:** 10.3389/fpsyt.2022.838535

**Published:** 2022-04-25

**Authors:** Shaikh I. Ahmad, Emily W. Shih, Kaja Z. LeWinn, Luisa Rivera, J. Carolyn Graff, W. Alex Mason, Catherine J. Karr, Sheela Sathyanarayana, Frances A. Tylavsky, Nicole R. Bush

**Affiliations:** ^1^Department of Psychiatry and Behavioral Sciences, University of California, San Francisco, San Francisco, CA, United States; ^2^Department of Anthropology, Emory University, Atlanta, GA, United States; ^3^College of Nursing, The University of Tennessee Health Science Center, Memphis, TN, United States; ^4^Center on Developmental Disabilities, The University of Tennessee Health Science Center, Memphis, TN, United States; ^5^Department of Preventative Medicine, The University of Tennessee Health Science Center, Memphis, TN, United States; ^6^Department of Pediatrics, University of Washington, Seattle, WA, United States; ^7^Department of Environmental and Occupational Health Sciences, University of Washington, Seattle, WA, United States; ^8^Center for Child Health, Behavior and Development, Seattle Children's Research Institute, Seattle, WA, United States; ^9^Department of Pediatrics, University of California, San Francisco, San Francisco, CA, United States

**Keywords:** prenatal stress, executive functioning, externalizing behavior, parenting, child psychopathology

## Abstract

**Objective:**

Experiences of stress and adversity, such as intimate partner violence, confer risk for psychiatric problems across the life span. The effects of these risks are disproportionately borne by women and their offspring—particularly those from communities of color. The prenatal period is an especially vulnerable period of fetal development, during which time women's experiences of stress can have long-lasting implications for offspring mental health. Importantly, there is a lack of focus on women's capacity for resilience and potential postnatal protective factors that might mitigate these intergenerational risks and inform intervention efforts. The present study examined intergenerational associations between women's prenatal stressors and child executive functioning and externalizing problems, testing maternal parenting quality and child sex as moderators, using a large, prospective, sociodemographically diverse cohort.

**Methods:**

We used data from 1,034 mother-child dyads (64% Black, 30% White) from the Conditions Affecting Neurocognitive Development and Learning in Early Childhood (CANDLE) pregnancy cohort within the ECHO PATHWAYS consortium. Women's prenatal stressors included stressful life events (pSLE) and intimate partner violence (pIPV). Measures of child psychopathology at age 4–6 included executive functioning and externalizing problems. Parenting behaviors were assessed by trained observers, averaged across two sessions of mother-child interactions. Linear regression models were used to estimate associations between women's prenatal stressors and child psychopathology, adjusting for confounders and assessing moderation effects by maternal parenting quality and child sex.

**Results:**

Women's exposures to pSLE and pIPV were independently associated with child executive functioning problems and externalizing problems in fully-adjusted models. Maternal parenting quality moderated associations between pSLE and both outcomes, such that higher parenting quality was protective for the associations between women's pSLE and child executive functioning and externalizing problems. No moderation by child sex was found.

**Discussion:**

Findings from this large, sociodemographically diverse cohort suggest women's exposures to interpersonal violence and major stressful events—common for women during pregnancy—may prenatally program her child's executive functioning and externalizing problems. Women's capacity to provide high quality parenting can buffer this intergenerational risk. Implications for universal and targeted prevention and early intervention efforts to support women's and children's wellbeing are discussed.

## Intergenerational Transmission of Effects of Women's Stressors During Pregnancy: Child Psychopathology and the Protective Role of Parenting

Exposure to various forms of stressors, including economic precarity, housing insecurity, loss of a loved one, and interpersonal violence, are well-established predictors of psychiatric problems across the life span (1–5). The impact of these experiences is disproportionately borne by women—especially women in underserved communities of color (6–9) and with lower incomes (10)—which also places their offspring at increased risk for later psychiatric problems. Indeed, the World Health Organization considers intimate partner violence against women a “major public health problem and a violation of women's human rights,” estimating that roughly one in three women are subjected to intimate partner violence during their lifetime, with up to 13% of women experiencing intimate partner violence during pregnancy (pIPV) (11). Further, the most common form of violence committed against women is intimate partner violence, and women who experience partner violence are at increased risk for a range of mental health problems, such as depression, posttraumatic stress, anxiety, and suicidality (11). In addition, according to the Centers for Disease Control and Prevention's Pregnancy Risk Assessment Monitoring System, in 2010 about 70% of pregnant women experienced at least one stressful life event (pSLE) in the year before their infant's birth (12). These include: emotional stressors (e.g., a family member being hospitalized or dying), financial stressors (e.g., moving, losing a job, being unable to pay rent/bills), partner-related stressors (e.g., separation/divorce), and traumatic stressors (e.g., becoming homeless) (12). Despite these alarming rates of exposure to stressors during pregnancy—a time when women's social and biological wellbeing is in flux and particularly vulnerable to stress (13, 14)— there has been limited focus on the intergenerational transmission of the effects of stressors, such as pSLE and pIPV, on offspring psychopathology and related developmental processes. The perinatal period is a critical time for offspring development, wherein such intergenerational risks pose a threat to offspring mental health across the lifespan. Crucially, there is also a lack of focus on women's capacity for resilience and associated research on potential postnatal resilience-promoting factors that might mitigate these intergenerational risks for child mental health problems and shed light on opportunities to support maternal and child wellbeing after exposure to prenatal adversity in affected communities.

A large body of research has documented the association of both pSLE and pIPV with deleterious outcomes for women, including maternal mortality, labor and delivery complications, poor perinatal mental health (such as depression, post-traumatic stress disorder, and substance use disorder), and enduring alterations to women's immune function (13, 15, 16). The intergenerational impact of pIPV and pSLE is seen in neonatal outcomes of higher rates of preterm birth and low birth weight, but also in longer-term neurodevelopmental problems that extend into childhood and beyond (17–20). Investigations of maternal IPV exposure are especially crucial considering the established continuity of IPV within families and across generations. For example, women who witness IPV as children have greater odds of experiencing IPV as adults, and their own children are more likely to witness IPV (21, 22). Children exposed to IPV also have increased risk for both externalizing and internalizing problems, as well as higher levels of symptoms of trauma, compared to non-exposed children (15, 23). Women's exposure to stressors in pregnancy might affect offspring development through both pre- and post-natal pathways, with potentially cumulative effects, making it important to expand empirical understanding of these risks.

Prenatal programming of offspring neurodevelopment and psychopathology in the context of maternal prenatal stress is a complex biopsychosocial phenomenon that requires attention to the social dynamics and biology that is unique to women. Women's experiences of stress during pregnancy result in altered fetal exposure to maternal glucocorticoids, immune tolerance, and nutrient supply that can shift trajectories of offspring growth and stress reactivity in the postnatal environment (24–27). Despite the fact that prenatal stressors such as pIPV and pSLE for women can co-occur, their dual contributions to child psychopathology are rarely studied—especially in communities of color (17, 28, 29). This is a particularly important population within which to examine such stressors given that women of color have higher prevalence rates of intimate partner violence compared to non-Hispanic White women (6–9).

Studies examining the intergenerational associations between maternal prenatal stress and risk for offspring psychopathology during infancy and early childhood have found that maternal prenatal stress is generally linked to her child's subsequent inability to self-regulate, manifesting as more difficult temperaments and increased stress reactivity (17, 30). These problems with self-regulation during early school-ages (i.e., preschool and kindergarten) can manifest as executive functioning (EF) deficits, which are also closely related to externalizing psychopathology, such as ADHD (31, 32). Given the high prevalence rates of EF problems and externalizing behavior in young children (33, 34) and how they set the stage for later, more severe psychopathology and health risk (35, 36), examining their etiology and development in early childhood is critical. Although some studies have found associations between prenatal maternal stress and offspring externalizing psychopathology, including risk for ADHD (28), fewer studies have examined this potential link between prenatal stress and child EF problems (37, 38), and to our knowledge, none have examined both executive functioning and externalizing problems within the same conceptual model. Importantly, very few studies have prospectively examined these associations in large, sociodemographically diverse samples, which is particularly salient given that lower SES places both women and their offspring at increased risk for psychopathology, in part due to greater exposure to stressors across generations (17, 18, 39).

There is evidence regarding differential sex effects of maternal stress on offspring psychopathology; however, to date these findings have been quite mixed—necessitating additional inquiry (17, 33, 40–43). This is particularly important to examine in outcomes with well-established sex differences, such as externalizing behavior problems during childhood. Sex-dependent differences in offspring responses to prenatal stress are complex biopsychosocial phenomena, wherein socially patterned norms for gendered child behavior interact with developmental psychobiology. Female and male fetuses exhibit sexually dimorphic responses to the maternal stress-related hormonal and cytokine milieu, investing differentially in placental and somatic growth in ways that may confer sex-dependent trajectories of buffering and risk in the wake of prenatal adversity (41, 44). While female fetal buffering from maternal inflammation or nutritional stress may confer increased resilience in terms of viability relative to males, trade-offs in increased sensitivity to HPA axis programming and risk for psychopathology may also occur (45, 46). For example, Graham and colleagues (47) found that elevated cortisol levels during pregnancy predicted increased amygdala and default mode network connectivity and mediated increased internalizing symptoms in 24-month-old girls but not boys. There is some evidence that boys might be more likely to develop externalizing problems during childhood within the context of maternal prenatal stress (40, 48, 49). Yet, findings here are still mixed, and fewer studies have examined these potential sex differences in early childhood, when externalizing problems are less sex differentiated (43, 47, 50).

Although research on integrational transmission of stress effects has burgeoned recently, there remains a dearth of research identifying opportunities for intervention or prevention regarding prenatal stress and risk for offspring psychopathology (17, 51–53). Identifying malleable, postnatal environmental factors that capitalize on women's strengths is also critical to reducing potential intergenerational risk (19). Parenting is certainly a key factor, with a wealth of research indicating that it can serve as both a risk and a protective factor for child mental health. For example, negative aspects of parenting, such as harsh discipline, neglect, and punishment, are associated with increased risk for offspring psychopathology—especially externalizing behavior problems (54, 55). In addition, multiple studies have found that prior exposure to IPV has a negative impact on parenting behaviors (56), and that such parenting behaviors are also associated with child behavior problems (57–59). However, the increased focus on the negative aspects of parenting has perhaps overshadowed the benefits that the positive aspects of parenting (such as warmth, responsiveness, and scaffolding) can have on offspring mental health. Indeed, positive parent-child interactions have been shown to confer beneficial effects on executive functioning and externalizing psychopathology in both observational and intervention studies (39, 60–62), and positive parenting has been identified as an important resilience-promoting factor for children at increased risk for psychopathology (63). Sensitive caregiving in the context of ongoing stressors is contingent on a caregiver's ability to harness emotional, cognitive, and material resources, especially when caregiving demands are high, such as when children are young (64). Notably, research findings emphasize the ability of caregivers to buffer children from adversity and support healthy child development through supportive parenting, even when facing socioeconomic barriers and other high-adversity contexts (65). For example, Narayan and colleagues found that women with high levels of childhood trauma and positive memories of nurturing care were able to buffer their children from future intergenerational trauma exposure (66). In another example, effective parenting and parental use of positive coregulation skills were associated with higher child executive functioning skills and had positive benefits at school among families who were experiencing homelessness (67). Most studies examining the associations between women's parenting behaviors and child psychopathology, however, rely on parent self-report of their own parenting behaviors, which can produce biased results (68, 69). Fewer studies use more objective, observer ratings of mother-child interactions and parenting behaviors which are less influenced by such biases.

The present study examined the intergenerational association between maternal prenatal stress and risk for executive functioning and externalizing behavior problems during early childhood (ages 4–6) in a large, prospective, pregnancy cohort study of mother-child dyads. We had three main aims to address gaps in extant literature. First, we tested whether women's stressors during pregnancy, including exposure to multiple types of pSLE and pIPV, were predictive of two key aspects of self-regulation and psychopathology in their children: EF and externalizing behavior problems. Importantly, we utilized a large, racially diverse (64% Black, 30% White) sample with a broad representation of lower-income families, characteristic of an urban Southern metropolitan area in the United States—a population that is largely understudied in extant literature and, due to structural inequalities, are likely to be exposed to higher levels of stressors during pregnancy (10, 70, 71). Second, we tested whether observer-rated parenting behaviors might serve as a postnatal environmental factor that moderates the association between prenatal stressors and risk for child psychopathology. Finally, given the potential for differential effects of prenatal stress on male vs. female offspring, we examined whether child sex moderated the effects of women's pregnancy stressors on both child outcomes.

## Materials and Methods

### Participants and Procedure

The present study utilized data from the Conditions Affecting Neurocognitive Development and Learning in Early Childhood (CANDLE) study, which is part of the ECHO PATHWAYS consortium (72, 73). CANDLE is a longitudinal pregnancy cohort study that enrolled 1,503 women from Memphis/Shelby County, Tennessee, between 2006 and 2011 during their second trimester of pregnancy. Women were between ages 16–40, did not have pre-existing chronic conditions that required medication, and had low-risk pregnancies. Overall, the sample was racially diverse (64% Black, 30% White) and, although the sample had a broad range of socioeconomic status, it was predominantly low-income (59% having federal or state-supplemented health insurance)—representative of the urban area from which it was drawn. All women provided informed consent and the study was approved by the University of Tennessee Health Science Center Institutional Review Board.

Baseline data were collected at study enrollment during the women's second trimester of pregnancy. Subsequent perinatal data were collected during a third trimester visit and at childbirth. Families were then prospectively followed, with data collected at a home visit 4-weeks postpartum, a 6-month phone follow-up, and subsequently at multiple clinic visits occurring at approximately child ages 1-, 2-, 3-, 4–6-, and 8-years. The final analytic sample comprised 1,034 women for whom child data were available on at least one outcome measure. Compared to the total enrolled sample of 1,503, women in the analytic sample tended to be older at study enrollment (*t* = 3.94, *p* < 0.001), but did not otherwise significantly differ on study variables.

### Measures

#### Prenatal Predictors

##### Stressful Life Events

Women reported retrospectively on whether they experienced 14 different types of major pSLE during pregnancy, using a measure adapted from the widely used Centers for Disease Control and Prevention Pregnancy Risk Assessment Monitoring System survey (74), within the age eight visit maternal questionnaire. This scale included the following 14 items: a family member was hospitalized; death of a close friend/family member; moving to a new address; loss of job/employment; partner lost their job; participant/partner had a reduction in work hours or pay; problems paying the rent/mortgage or other bills; separation/divorce from partner; was apart from partner due to military deployment or extended work-related travel; argued with partner more than usual; partner did not want participant to be pregnant; close friend/family member had a problem with drinking/drugs; participant/partner was incarcerated; participant was homeless. Women responded yes or no to each item; all responses were summed into a total count of different types of pSLE experienced (range 0–14). Given the magnitude and significance of these stressful life events, such measures are thought to have limited recall bias and be accurate over a span of years (75, 76).

##### Intimate Partner Violence

Women reported on their experiences of pIPV via the short-form version of the revised Conflict Tactics Scale (77), which assesses multiple forms of partner violence. Information was collected during the third trimester of pregnancy, wherein women indicated if they experienced any of four different forms of partner violence (including physical, sexual, or emotional abuse, and/or injury) during the past year. Each item (answered yes or no) was summed to create a total pIPV score (range 0–4).

### Moderators

#### Parenting Quality

Parenting behaviors were assessed at both the age 2 and age 3 clinic visits using the Nursing Child Assessment Satellite Training (NCAST) Parent-Child Interaction Teaching Scale (78, 79). The NCAST comprises 73 items, each endorsed yes or no. This form was completed by study staff members who received rigorous training in the NCAST coding system, and was filled out by a staff member immediately after observing interactions between the mother and child as the mother teaches her child a developmentally challenging task (80). This measure has been utilized with diverse populations and has shown good internal consistency and test-retest reliability. Cronbach's alphas for the present sample were also good (α = 0.83 for Total Caregiver score and α = 0.81 for Total Caregiver-Child score) (81). We use the term “parenting quality” to describe the overall measure of parenting behavior captured by the Total Caregiver score. This comprises a range of parenting behaviors, including social, nurturant, and didactic caregiving (81). The Total Caregiver score consists of four subscales, including: parental sensitivity to cues, response to distress, social-emotional growth-fostering, and cognitive growth-fostering. Higher scores indicate more sensitivity, supportiveness, and scaffolding by mothers during observed interactions with their child. We created a composite parenting quality score across both the age 2 and age 3 clinic visits by averaging the Total Caregiver score across both visits. Data from one visit was used if both visit data were not available.

#### Child Sex

We tested the potential moderating effects of child biological sex, ascertained from birth records, on both outcome variables, given the mixed evidence regarding potential differences from previous research (17, 40).

### Child Outcomes

Child psychopathology was assessed using two measures at the age 4–6 clinic visit.

#### Executive Functioning Problems

Women reported on their children's EF using the Behavior Rating Inventory of Executive Functioning–Preschool version (BRIEF-P) (82) at the age 4–6 clinic visit. The BRIEF is a widely used measure that assesses a broad range of executive functioning problems in everyday life and is used in clinical and research settings. The BRIEF-P comprises 3 indexes—inhibitory self-control, flexibility, and emergent metacognition, which make up the overall global executive composite. Cronbach's alpha for the present sample was excellent (α = 0.96). The present study utilized *t*-scores of the overall composite to assess problems with executive functioning.

#### Externalizing Problems

Women reported on their children's externalizing problems via the well-validated and widely-used Child Behavior Checklist for ages 1.5–5 (CBCL) (83) at the age 4–6 clinic visit. Consistent with prior research, we used *t*-scores from the broadband Externalizing Problems scale, which has been widely used to assess overall problems of externalizing psychopathology, including hyperactivity/impulsivity, self-regulation, oppositionality, conduct, and aggression in children. Internal consistency for the Externalizing Problems scale was excellent (Cronbach's α = 0.91).

### Covariates

Several pre- and postnatal covariates were included to address potential confounding. We included several socioeconomic factors, given they have demonstrated associations with child psychopathology. The following were obtained from women during study enrollment: age, annual household income (adjusted for number of dependents), education, marital status, and self-reported race [the authors acknowledge that race is not a biological variable and is a political and social construct that often serves as a proxy for the impact of racist practices and structural inequality (84); thus, it is examined in the current paper with this premise in mind]. We also included women's full-scale IQ from the Wechsler Abbreviated Scale of Intelligence, Second Edition (85) assessed at the postnatal 1-year clinic visit, or thereafter if that visit was missed. Given that parent psychopathology, in particular depression, is also associated with later child psychopathology, we included both postpartum and concurrent maternal depression as covariates. Women's postpartum depression across the 1st year of the child's life was measured with the 10-item, self-reported Edinburgh Postnatal Depression Scale (86), which was assessed at 4-, 6-, and 12-months post-birth; the total depression score across all three time points was averaged into a single composite. Concurrent maternal depression was measured at the age 4–6 clinic visit with the Center for Epidemiologic Studies Depression Scale, a 20-item self-report (87). Finally, child biological sex and age at the outcome timepoint were included as covariates in all models.

### Statistical Analyses

All linear regression models were conducted using R (RStudio version 1.2.5033) and fitted using the *lm* function. Data were first examined for normality and for the presence of outliers in study variables. The missForest package (88) was used to impute missing data using the random forest multiple imputation method. In comparison with other multiple imputation methods (e.g., MICE), this machine learning technique can accommodate non-linearities and interactions and does not need a specific regression model to be specified, which makes this approach more useful for imputing larger data sets where some participants have missing data (89). All variables had missing data of 5% or less except for maternal pSLE, which was missing for 22%. Linear regressions were conducted to test hypotheses using the imputed data set. All predictor variables and covariates were standardized before being entered into the models. The following analyses were conducted in two separate models, one for each outcome variable (EF and externalizing problems).

In Step 1, we estimated main effects by performing multiple linear regressions to examine the relation between both prenatal predictors (pSLE, pIPV) and each outcome variable without including covariates in the model. In Step 2, we added all covariates to the models in Step 1 to obtain our fully-adjusted models. In Step 3, we examined potential interactions with parenting quality by additionally incorporating the two interaction terms between each prenatal predictor and the parenting quality moderator (pSLE x parenting quality; pIPV x parenting quality) into both of the fully-adjusted models for our two outcome variables. Significant interaction terms were then probed to test for simple slopes at three different values of the moderator (+1 SD, mean, −1 SD) (90). Finally, we repeated the same procedures to examine potential interaction effects of child sex by prenatal predictors on both outcome variables by including interaction terms between each prenatal predictor and child biological sex.

## Results

Descriptive statistics of the primary study variables are presented in Table 1. Median education level was completion of high school; mean household-adjusted income was $18.4 k. Regarding pSLE, 28% of women reported experiencing no pSLE; 50% reported experiencing at least 1 type of pSLE; 32% reported experiencing at least 2; and 21% reported experiencing 3 or more. For pIPV, 28% of women reported experiencing no forms of pIPV; 66% reported experiencing at least 1 form; 18% reported experiencing at least 2; and 7% reported experiencing 3 or more forms of pIPV. Thirty four percent of women reported experiencing both pIPV and pSLE. Children's EF problems were strongly correlated with externalizing problems (*r* = 0.7, *p* < 0.001). Bivariate correlations are provided in Table 2. Of note, maternal pSLE and pIPV were weakly correlated (*r* = 0.19, *p* < 0.001), suggesting they captured largely unique domains of stress exposure. Also of note, observed parenting quality was neither correlated with pSLE (*r* = −0.03, *p* = 0.49) nor pIPV (*r* = −0.05, *p* = 0.16), suggesting the consideration of parenting as a potential moderator, rather than a mediator, was appropriate.

**Table 1:** Demographic information and model variables (*N* = 1,034).

**Variable**	**Characteristics (range)**	**Mean (SD)** **or *n* (%)**
Maternal variables		
Age (years)	Age at study enrollment	26.4 (5.6)
Education	Some elementary/high school	113 (10.9)
	Graduated high school/GED	493 (47.6)
	Graduated technical school	98 (9.5)
	Bachelor's degree	209 (20.2)
	Graduate/professional degree	121 (11.7)
Partner status	Married/living with partner	579 (56.0)
	Single/divorced/not married	454 (43.9)
Adjusted household income	Adjusted for household size	$18.4 k ($17.0 k)
Race	Black	661 (63.9)
	White	308 (29.8)
	Other	65 (6.3)
Postnatal depression	Edinburgh Postnatal Depression Score (0–22)	4.4 (3.6)
Concurrent depression	Center for Epidemiologic Studies Depression (0–49)	8.5 (7.1)
Intimate partner violence	Conflict Tactics Scale (0–4)	0.9 (0.88)
Stressful life events	PRAMS SLE (0–14)	1.7 (1.9)
Parenting quality	NCAST Total Caregiver scale (19–50)	39.7 (5.3)
Child variables		
Age (years)	Age at 4–6 year clinic visit	4.3 (0.4)
Sex	Female	519 (50.2)
	Male	515 (49.8)
Externalizing problems	CBCL T-score (28–88)	44.8^a^ (10.3)
Executive functioning	BRIEF-P T-score (33–104)	47.9^b^ (11.3)
problems		

a *8% of children had T-scores that fell within or above the borderline range on the CBCL.*

b *9% of children had T-scores that fell in the clinically significant range on the BRIEF-P*.

**Table 2:** Correlations between study variables.

	**1**	**2**	**3**	**4**	**5**	**6**	**7**	**8**	**9**	**10**	**11**
1. Maternal age	1										
2. Child age	−0.14***	1									
3. Adjusted annual income	0.49***	−0.09*	1								
4. Maternal full-scale IQ	0.42***	−0.08*	0.60***	1							
5. Postnatal depression	−0.01	0.04	−0.08*	−0.03	1						
6. Concurrent depression	−0.10**	0.02	−0.21***	−0.16***	0.39***	1					
7. Prenatal SLE	−0.15***	−0.03	−0.23***	−0.04	0.19***	0.22***	1				
8. Prenatal IPV	−0.08*	0.05	−0.14***	−0.07	0.17***	0.10**	0.19***	1			
9. Parenting quality	0.35***	−0.11**	0.43***	0.52***	0.04	−0.11**	−0.03	−0.05	1		
10. EF problems	0.01	0.04	−0.04	−0.01	0.26***	0.30***	0.13**	0.14***	−0.07	1	
11. Externalizing problems	0.00	−0.05	−0.04	−0.01	0.26***	0.29***	0.19***	0.14***	−0.08*	0.70***	1
Sample size per variable^a^	1,034	1,034	1,030	1,022	1,004	1,024	799	974	977	1,024	1,030

*EF, Executive Functioning; IPV, Intimate partner violence; SLE, Stressful life events.*

a *Analytic dataset N = 1,034.*

* *p < 0.05*,

** *p < 0.01*,

*** *< 0.001*.

Tables 3, 4 present results from the regression analyses, examining both main and moderated effects, for child EF problems and externalizing problems, respectively. Results for the fully-adjusted model for child EF problems (Table 3) showed maternal pSLE (b = 1.13, *p* = 0.004) and pIPV (b = 1.02, *p* = 0.004) independently predicted child EF problems, such that higher levels of pSLE and pIPV were uniquely, positively associated with levels of child EF problems. There was a marginally significant main effect of observed parenting quality on child EF problems, wherein higher levels of parenting quality were associated with lower levels of EF problems in childhood (b = −0.81, *p* = 0.044). Regression results for the fully-adjusted model for child externalizing problems (Table 4) similarly showed women's pSLE (b = 1.30, *p* < 0.001) and pIPV (b = 0.94, *p* = 0.003) independently predicted child externalizing problems, such that higher levels of pSLE and pIPV were uniquely, positively associated with levels of child externalizing problems. In addition, higher levels of parenting quality were significantly associated with fewer child externalizing problems (b = −1.11, *p* = 0.003). Of note, women's postpartum depression and concurrent depression independently predicted both child outcomes.

**Table 3:** Regression models of maternal prenatal stress and child executive functioning.

**Model**	**Predictors**		***B***	**95% CI**	**SE**	***P***
Step 1:	Prenatal predictors					
Predictors only^a^	Prenatal SLE		1.78	[1.01, 2.56]	0.40	<0.001***
	Prenatal IPV		1.43	[0.72, 2.14]	0.35	<0.001***
Step 2:	Covariates					
Full model^b^	Maternal age		0.71	[−0.12, 1.53]	0.42	0.093
	Adjusted household income		0.26	[−0.78, 1.31]	0.53	0.626
	Maternal race (Black)		4.96	[2.94, 6.97]	1.02	<0.001***
	Maternal full-scale IQ		−0.45	[−1.50, 0.60]	0.54	0.404
	Postpartum depression		1.38	[0.66, 2.11]	0.37	<0.001***
	Concurrent depression		2.69	[1.97, 3.40]	0.36	<0.001***
	Parenting quality		−0.83	[−1.52, 0.06]	0.40	0.044*
	Child age		0.54	[−0.11, 1.19]	0.33	0.106
	Child sex (female)		2.45	[1.17, 3.73]	0.65	<0.001***
	Prenatal predictors					
	Prenatal SLE		1.13	[0.35, 1.91]	0.40	0.004**
	Prenatal IPV		1.02	[0.33, 1.70]	0.35	0.004**
Step 3:	Moderators					
Interaction effects	SLE × Parenting quality		−0.98	[−1.74, −0.23]	0.38	0.010*
	IPV × Parenting quality		0.51	[−0.18, 1.19]	0.35	0.146

a *Predictors-only model accounted for 4% of variance in child EF problems.*

b *Fully-adjusted model accounted for 18% of variance in child EF problems.*

* *p < 0.05*,

** *p < 0.01*,

*** *p < 0.001*.

**Table 4:** Regression models of maternal prenatal stress and child externalizing problems.

**Model**	**Predictors**		***B***	**CI 95**	**SE**	***p***
Step 1:	Prenatal predictors					
Predictors only^a^	Prenatal SLE		1.94	[1.24, 2.64]	0.36	<0.001***
	Prenatal IPV		1.19	[0.54, 1.83]	0.33	<0.001***
Step 2:	Covariates					
Full model^b^	Maternal age		0.37	[−0.39, 1.13]	0.39	0.338
	Adjusted household income		0.30	[−0.66, 1.26]	0.49	0.536
	Maternal race (Black)		3.16	[1.31, 5.01]	0.94	<0.001***
	Maternal full-scale IQ		0.32	[−0.65, 1.28]	0.49	0.524
	Postpartum depression		1.12	[0.46, 1.79]	0.34	<0.001***
	Concurrent depression		2.26	[1.60, 2.92]	0.34	<0.001***
	Parenting quality		−1.11	[−1.84, −0.38]	0.37	0.003**
	Child age		−0.72	[−1.32, −0.12]	0.31	0.019*
	Child sex (female)		1.19	[0.02, 2.37]	0.60	0.047*
	Prenatal predictors					
	Prenatal SLE		1.30	[0.58, 2.02]	0.36	<0.001***
	Prenatal IPV		0.94	[0.31, 1.57]	0.32	0.003**
Step 3:	Moderators					
Interaction effects	SLE × Parenting quality		−0.75	[−1.45, −0.06]	0.35	0.034*
	IPV × Parenting quality		0.53	[−0.10, 1.16]	0.32	0.100

a *Predictors-only model accounted for 5% of variance in child externalizing problems.*

b *Fully-adjusted model accounted for 16% of variance in child externalizing problems.*

* *p < 0.05*,

** *p < 0.01*,

*** *p < 0.001*.

**Figure 1:**
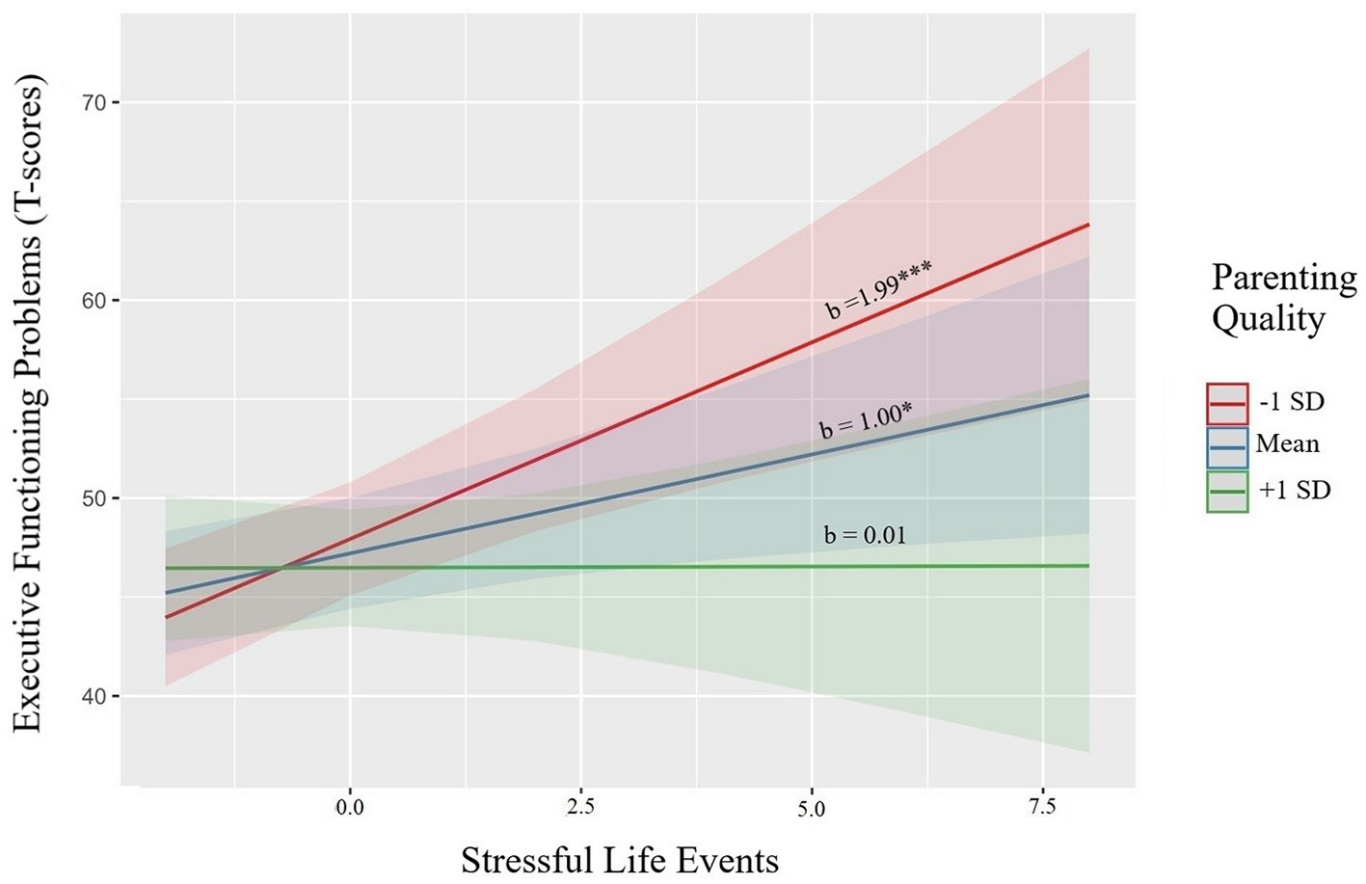
Parenting quality moderates the association between maternal prenatal stressful life events and child executive functioning problems.

Next, we examined whether parenting quality moderated the aforementioned associations. Two significant interactions emerged, qualifying the main effects found. Regarding child EF problems (Table 3, Step 3), there was a significant pSLE x parenting interaction (b = −0.98, *p* = 0.010). Figure 1 provides illustration of the continuous interaction term plotted, with tests of the simple slopes, at the mean and +/– 1 SD, showing a significant positive association between women's pSLE and children's EF problems at average (b = 1.00, SE = 0.40, *p* = 0.012) and at low levels (−1 SD; b = 1.99, SE = 0.53, *p* < 0.001) of observed parenting quality. However, at higher levels of observed parenting quality (+1 SD), there was a buffering effect such that pSLE was not significantly associated with child EF problems (b = 0.01, SE = 0.57, *p* = 0.985). Parenting quality did not significantly interact with pIPV to predict child EF problems (b = 0.51, *p* = 0.146).

**Figure 2:**
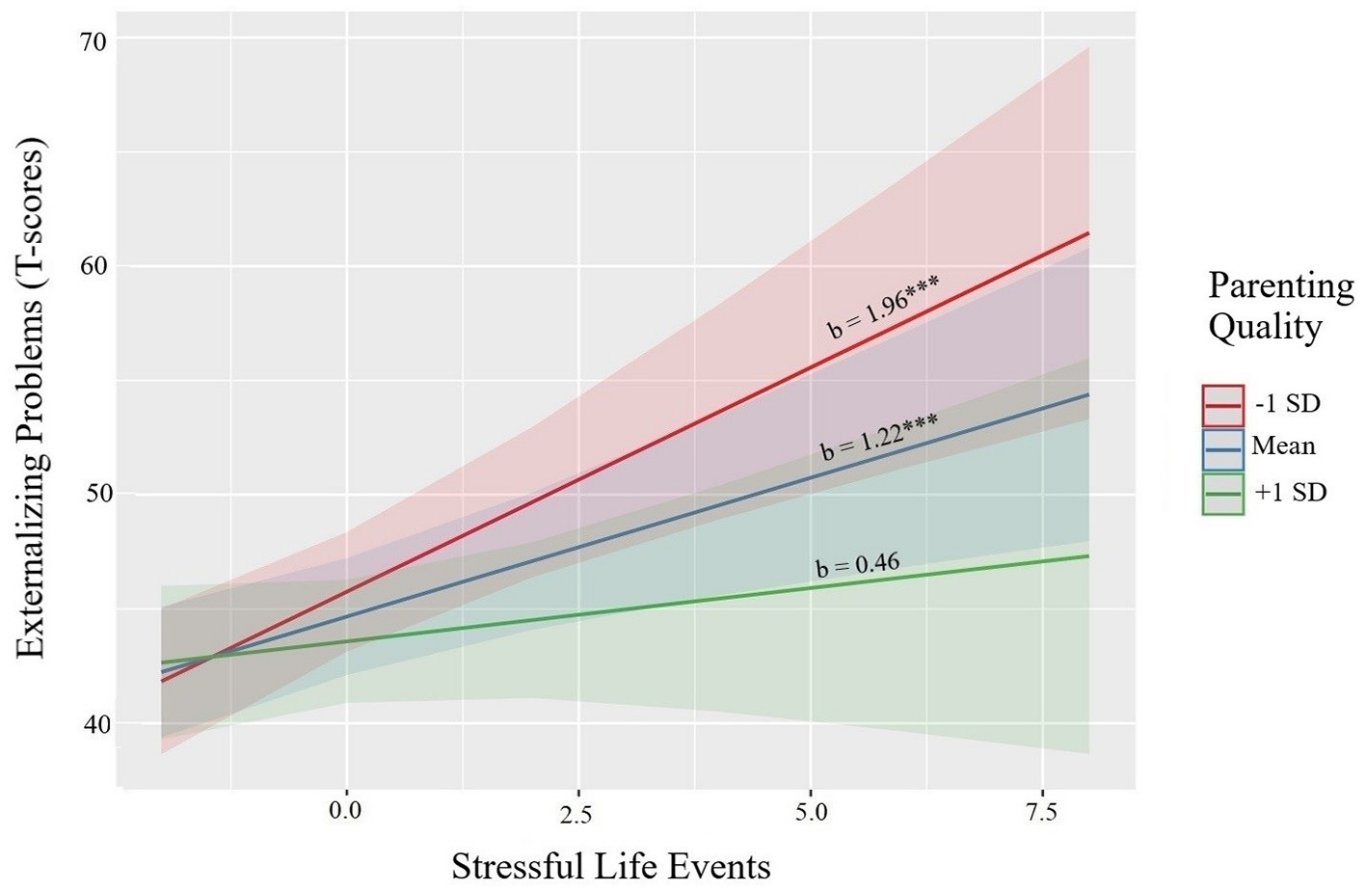
Parenting quality moderates the association between maternal prenatal stressful life events and child externalizing problems.

A similar pattern was found for child externalizing problems (Table 4, Step 3), revealing a significant pSLE × parenting interaction (b = −0.75, *p* = 0.034). Figure 2 provides illustration of the continuous interaction term plotted, with tests of the simple slopes, showing a significant positive association between women's pSLE and children's externalizing problems at average (b = 1.22, SE = 0.36, *p* < 0.001) and low levels (−1 SD; b = 1.96, SE = 0.49, *p* < 0.001) of parenting quality. At higher levels of observed parenting quality, there was again a buffering effect, such that pSLE was not significantly associated with child externalizing problems (b = 0.46, SE = 0.52, *p* = 0.373). There was no significant interaction between pIPV and parenting quality for child externalizing problems (b = 0.53, *p* = 0.100). Finally, we examined associations between child sex and both measures of women's pregnancy stress exposure, predicting both outcomes. Although girls displayed lower problems in adjusted models for both outcomes, there was no evidence for moderation by child sex for either stress exposure (results not shown).

## Discussion

Understanding the complex biopsychosocial phenomenon of relations between women's exposure to stressors in pregnancy and child psychopathology is important for the prevention of mental illness and the promotion of women and children's wellbeing. The aim of the present study was to examine the intergenerational associations between women's stress exposures during pregnancy and young childhood executive functioning and externalizing problems in a large, sociodemographically diverse sample. Crucially, we also examined women's observed parenting quality as a potential postnatal protective factor that might buffer children from the risks of later psychopathology. To our knowledge, this is one of the largest U.S. pregnancy cohorts examining intergenerational associations of maternal prenatal stress exposures and child psychopathology that includes a large percentage of Black women as well as a broad representation of families with lower household income—a sample with particular generalizability to Southern metropolitan U.S. populations. We found that women's exposure to intimate partner violence and stressful life events during pregnancy independently predicted higher levels of EF and externalizing problems in their 4–6-year-old children, even after controlling for a variety of pre- and post-natal factors. Notably, these two prenatal stress exposures were very weakly correlated, suggesting that different domains of women's risk exposure during pregnancy have unique relevance to child development and psychopathology. In addition, we found that higher levels of sensitivity, supportiveness, and scaffolding provided by women to their children during observed parent-child interaction tasks served as a postnatal protective factor, buffering their children from the association between pSLE and both child outcomes—providing insights into heterogeneity of main effects and potential targets for intervention.

Our findings for the associations between women's prenatal stressors and child externalizing problems are largely consistent with extant literature (17, 28), yet they expand the evidence base to EF outcomes within a large, sociodemographically diverse sample. Although the operationalization of child EF across the few existing studies has varied (37, 38), our findings—which utilized a behaviorally-based measure of EF—are generally consistent with the few prenatal programming studies that have used lab-based measures of EF, indicating that women who experienced more forms of stress during pregnancy reported that their children demonstrated poorer overall inhibition, flexibility, planning and/or working memory in everyday settings. Given the strong association between child EF and externalizing problems, the fact that we see a similar pattern in prediction by both prenatal stressors is not necessarily surprising and further suggests shared etiology or developmental mechanisms (91). In addition, our finding regarding the negative associations between maternal parenting quality and child EF and externalizing problems is also consistent with a large body of extant research on parenting and child behaviors. Women who have the social/emotional/economic resources, structural supports, and ability to provide more scaffolding, warmth, and nurturing to their children are more likely to help mitigate and reduce potential externalizing behavior problems. Interventions that support a caregiver's ability to harness resources for supportive parenting may thus play a crucial role in mitigating the impact of early adversity in children most at risk. Importantly, although parenting behaviors can certainly be shaped by prior experiences of stress and adversity, we found that neither stress exposure was associated with observer-rated postnatal parenting behaviors in the present study, suggesting that these parenting behaviors were not influenced by women's experiences of stressful life events or intimate partner violence during pregnancy.

A robust literature documents the effects of the postnatal environment on child mental health, including stressors within the home, parental psychopathology, and parenting behaviors (70, 92–96). Accordingly, the present study accounted for many of these in our models—including several socioeconomic, psychosocial, and environmental factors—though this was not an exhaustive list. Although a growing body of work also documents the intergenerational associations between the prenatal environment and offspring psychopathology, such prenatal stressors likely operate through multiple pre- and postnatal pathways (both psychobiological and psychosocial). Findings presented herein should be interpreted within this context. Indeed, our findings suggest that these main effects of prenatal stressors should be considered within the context of the postnatal caregiving environment. Our study makes a particularly novel contribution in demonstrating that multi-year assessments of observer-rated maternal parenting behaviors moderated the association between women's pSLE and both child EF and externalizing problems. Specifically, for women who were able to provide higher levels of scaffolding, support, and encouragement to their children across several parent-child interaction tasks spanning two assessment time points, there was no increased risk of later child psychopathology associated with higher levels pSLE. This is contrasted with women who might not have had the necessary resources, structural supports, and ability to provide the same levels of scaffolding and encouragement to their children, wherein there was an increased risk of both later child EF and externalizing problems associated with higher levels of pSLE. These findings also suggest that many individuals are capable of providing high quality parenting despite prior exposure to adversity and violence (14, 66, 97). Interestingly, this moderating pattern was not found for prenatal intimate partner violence and either measure of child functioning. Methodology may be a possible explanation, given the difference in the range of possible values for each measure of maternal prenatal stress. Whereas, the pSLE measure had a range of 0–14, the measure for pIPV only had a range of 0–4, which might have reduced the ability to detect variations in scores between study participants based on differences in parenting quality. It is also possible that women's violence exposure, specifically, has less heterogeneous effects on offspring.

The present study highlights the importance of supporting and fostering women's capacity to optimize the caregiver-child relationship—often ignored in the prenatal programming literature—which served as a potential resilience-promoting factor in the prediction of child mental health problems. Women's capacity to provide sensitive, supportive parenting is not simply the product of—or responsibility of—a single caregiver, but rather a reflection of the larger community and societal context within which that parenting occurs (98). Many social determinants that affect maternal or child health and wellbeing also affect children's rearing environments, and therefore the resources that parents and caregivers have available to them within a given environment. Viewed in that framework, it is not surprising that community-based parenting programs that promote caregiver self-care, connection to resources, and knowledge of child attachment have proven efficacious in communities affected by violence (99, 100). Beyond community-based interventions that focus on providing more supports to parents and caregivers in higher-risk settings, national policies that increase resources for pregnant women and other caregivers—such as expansion of perinatal Medicaid coverage, increased parental leave, and perinatal cash transfer programs—can uplift our capacity to break intergenerational cycles of risk for child psychopathology and poor health (101–103). A growing body of literature highlights the importance and benefits of offering universal access to evidence-based parenting support and training programs—especially early in child development (104). In addition to improving child functioning, such intervention programs have also been shown to improve women's mental health (105). Indeed, multiple studies have shown that interventions focused on either parents or the parent-child relationship among families exposed to IPV, for example, have positive effects for both mothers and children (106, 107). Further, providing such universal access at a population level would not only allow all families to benefit from such programs but would also help in destigmatizing them.

Finally, regarding our test of moderation by child sex, we did not find that child sex significantly moderated the association between women's prenatal stressors and child psychopathology—although our sample was likely sufficiently powered to do so. This is not necessarily surprising, given the mixed findings regarding sex differences in previous examinations (17, 40). Indeed, although there was a main effect of child sex, wherein, on average, girls displayed marginally lower levels of EF problems, and significantly lower levels of externalizing problems, compared to boys—consistent with prior literature—these associations were not moderated by maternal stressful life events or intimate partner violence during pregnancy.

Also of note, fully one half of our sample experienced at least one type of pSLE (with almost one-third experiencing at least two), two-thirds reported experiencing at least one form of pIPV, and fully one third experienced at least one form of both pSLE and pIPV. These rates are in the higher range of estimates for pIPV prevalence, and may reflect unmet need for violence prevention and perinatal adversity support for women in this population (6, 8). Social inequity in the greater Memphis/Shelby County, Tennessee area—from which the present study cohort was recruited—has been associated with poor child health and educational outcomes, yet our findings also indicate the need for attention to upstream perinatal prevention and intervention efforts for families in this and similar populations (108–110). Further, we found that the associations between both pIPV and pSLE and child outcomes were independent of each other and, considered cumulatively, may have a greater impact on child mental health. Indeed, the stress exposures amounted to roughly 20–30% of the overall variance in psychopathology risk accounted for by each model.

The present study has a number of strengths: the use of a large, sociodemographically diverse sample including understudied Black urban Southern women, broad socioeconomic distribution across the sample, multiple indicators of prenatal stress exposure, and observer-rated parenting quality. However, there are several limitations. First, child outcome measures were reported by mothers. Recent work suggests limited bias from maternal report of child psychopathology (111), though we included important maternal covariates in our models to minimize potential reporter biases. Future studies would benefit from utilizing other informants and objective measures of child behavior. Second, although the inclusion of multiple domains of stressors, using two fairly distinct measures of exposures, is a strength—and adverse exposure counts are increasingly found to be strong predictors of health (112)—our measures did not consider the frequency or severity of the events, or the perceived experiences of distress from these stress exposures—all of which can contribute to the intergenerational effects of toxic stress on child functioning. Third, women retrospectively reported on pSLE when their children were ~8 years old. Although this approach is commonly used and recent evidence further supports validity of its use (especially for more significant and memorable life events) (75, 76, 113), there is still a potential for event recall bias. In addition, women's pIPV was assessed in the third trimester of pregnancy, wherein women reported their experiences over the past year, leaving the possibility that some pIPV was experienced in the few months prior to pregnancy. Finally, other sources of stress and adversity—both during pregnancy and postnatally—are relevant to offspring psychopathology (such as maternal experiences of daily stressors, racism, discrimination, as well as offspring exposure to traumatic events during childhood) but were not assessed with the present sample. Future intergenerational research would benefit from their inclusion.

Our findings add support to a growing body of research indicating the importance of *preventing* women's experiences of traumatic and stressful events during pregnancy—not just to protect women, but also for the potential intergenerational benefits with respect to offspring mental health. Moreover, our novel findings show that prenatal risks for child psychopathology are not uniform across families and may be buffered by strengthening and supporting the caregiving environment in the home. Interventions that provide additional support to families—both during the prenatal and postnatal period—and that include strategies and resources to strengthen caregiver-child relationships can play a key role in promoting caregiver and child resilience—even in the context of adversity (114). Indeed, caregiver wellbeing and behavior is key to promoting the development of child self-regulation skills, and could help prevent the development of later psychopathology (32, 54, 63). Further, given the transactional and cascading nature of the parent-child relationship—especially with regard to child externalizing psychopathology—ameliorating or preventing child mental health problems can also have downstream benefits of improving or preventing mental health problems for women who are mothers (115–117). Future research and intervention work, as well as health policy efforts, should focus on providing standard screening and universal preventative care to women during pregnancy (118) as a means of preemptively eliminating or reducing stressors for pregnant women and expecting families. Finally, additional research is certainly needed to identify more modifiable, postnatal resilience-promoting factors (19, 119, 120) in order to promote wellbeing across two generations and ameliorate risks for child psychopathology.

## Data Availability Statement

The original contributions presented in the study are included in the article/supplementary materials, further inquiries can be directed to the corresponding author.

## Ethics Statement

This study was reviewed and approved by the University of Tennessee Health Science Center Institutional Review Board. Written informed consent to participate in this study was provided by the participants and/or their legal guardian/next of kin.

## Author Contributions

NB, KL, CK, SS, and FT played a role in funding acquisition for the project supporting this manuscript. FT, JG, NB, and KL contributed to conception and design of the cohort study from which the data were drawn. FT was primarily responsible for project administration and supervision of data acquisition and curation, with support for data curation from NB and KL. SA and NB devised the manuscript study question. SA and ES designed the analytic approach for the study, with oversight by NB and wrote the first draft of the manuscript. ES performed the statistical analyses. LR and NB wrote sections of the manuscript. NB supervised the writing. All authors contributed to the interpretation of results, manuscript revision, and read and approved the submitted version.

## Funding

The ECHO PATHWAYS consortium was funded by the NIH (grants 1UG3OD023271-01 and 4UH3OD023271-03). The CANDLE study was also funded by the Urban Child Institute and the NIH (R01 HL109977). The present study was also affiliated with support from the CANDLE Developmental Origins of Health and Disease study (CIHR award number MWG-146331). NB is the Lisa and John Pritzker Distinguished Professor of Developmental and Behavioral Health and receives support from the Lisa Stone Pritzker Family Foundation and the Tauber Family Foundation.

## Conflict of Interest

The authors declare that the research was conducted in the absence of any commercial or financial relationships that could be construed as a potential conflict of interest.

## Publisher's Note

All claims expressed in this article are solely those of the authors and do not necessarily represent those of their affiliated organizations, or those of the publisher, the editors and the reviewers. Any product that may be evaluated in this article, or claim that may be made by its manufacturer, is not guaranteed or endorsed by the publisher.

## Abbreviations

EF: executive functioning
pIPV: prenatal intimate partner violence
pSLE: prental stressful life events.
